# Strength Characterization of Soils’ Properties at High Strain Rates Using the Hopkinson Technique—A Review of Experimental Testing

**DOI:** 10.3390/ma15010274

**Published:** 2021-12-30

**Authors:** Kamil Sobczyk, Ryszard Chmielewski, Leopold Kruszka, Ryszard Rekucki

**Affiliations:** Department of Military Engineering and Military Infrastructure, Faculty of Civil Engineering and Geodesy, Military University of Technology, 2 Gen. Sylwester Kaliski Str., 00-908 Warsaw, Poland; kamil.sobczyk@wat.edu.pl (K.S.); leopold.kruszka@wat.edu.pl (L.K.); ryszard.rekucki@wat.edu.pl (R.R.)

**Keywords:** high strain rate, soil, split Hopkinson pressure bar, review

## Abstract

The paper presents a review of crucial experiments and the latest publications, presenting the previous and current trends in experimental research in 2018–2021 in the area of soil dynamic interaction based on the Hopkinson bar technique. A review of investigated experimental test stands was made, in particular, cohesive and non-cohesive soil specimens prepared with different dimensions and densities. From this study, it can be concluded that the dynamic response of the soil depends on many factors, e.g., density, cohesion, moisture and grain structure of the soil specimen. There is still a noticeable interest in SHPB experiments performed in both 1D and 3D versions under modified conditions (frozen/heated soil specimen, different degree of water saturation content of the soil sample) in a wide range of strain rates 10^2^–10^4^ s^−1^, which is a large field for further research. The need to learn about the characteristics of various types of soil (both cohesive and non-cohesive) for the selection of structural design solutions for the protection elements of critical infrastructure was emphasized.

## 1. Introduction

Knowledge of both the static and dynamic behavior of the soil is important in geotechnical issues in building construction, in particular in the area of building foundations. The soil medium may be subjected to various static and dynamic loads. According to current reports, there are currently many threats to the safety of load-bearing structures of buildings, including the subsoil [1,2,3], in particular:

(I) Threats as a result of natural disasters—floods, landslides, storms, hurricanes, tornadoes, snowstorms;
(II) Threats related to intentional human activity—military and terrorist activities in the form of a bomb explosion, rocket attack, use of an explosive;
(III) Threats related to unintended human activity—accidents and collisions, construction errors of building structures, improper operation and maintenance of building structures.

Some of these threats (e.g., landslide, missile attack) are dynamic interactions and generate a soil strain rate of $10^{2}–10^{4}$ $s^{-1}$. Further behavior of the soil loaded in this way is of great importance for the maintenance of the foundation of the building and the possible consequences in the form of a construction disaster. Construction works can also have a dynamic effect on the surrounding buildings, e.g., dynamic soil compaction or driving of foundation piles. There is need to understand the characteristics of different types of soil (both cohesive and non-cohesive) in the best possible way in order to properly select design and construction solutions for buildings, protective elements and critical infrastructure. For this, a split Hopkinson pressure bar (SHPB) test stand is used. This apparatus allows one to determine the dynamic characteristics of the analyzed soil and to learn the stress–strain relationship, taking into account the strain rate for a known density, moisture and grain structure of the soil specimen. In the case of the procedure of determining the constitutive model of soil subjected to dynamic action, additional experiments are required to determine the necessary coefficients and frequencies.

The SHPB stand is designed, depending on its configuration, to test construction materials for dynamic effects—metal [4,5,6,7,8,9,10,11,12,13,14,15,16], concrete [17,18,19,20], wood, rocks [21,22,23,24,25,26,27,28,29], soil [30,31,32,33,34,35,36,37,38,39,40,41,42,43,44,45,46,47,48,49,50] and other materials such as polymers, ceramics, etc. [51,52,53,54,55,56,57,58].

The dynamic properties of the soils are also studied using various methods, for example, bent elements and a resonance column, offering a medium-to-high frequency spectrum capable of capturing the dynamic constitutive behavior of the soil [59].

In recent years, reviews and comparisons of studies for various strain rates have been made using the Hopkinson bar technique [60,61,62,63,64]. In the area of soil, significant reviews were carried out in 2012 [65] and 2015 [66]. The aim of this study was to make a compilation of the current crucial research trends in the area of soils tested with dynamic interaction induced by the Hopkinson bar technique.

## 2. Strain Rate Ranges

Over the last several decades, many experimental devices have been used to determine the characteristics of the reaction of the impact on materials through the degree of deformability at various ranges of strain rates. In both the military and commercial civil industries, the dynamic response of the material is a broad and crucial field for scientific analysis. Essentially, a general division can be made that materials deform differently under the action of static and dynamic loads. There are divisions presented in Table 1 according to the ranges of the strain rate and the corresponding experimental techniques. The data were compiled based on strain range analyses for various materials [67,68,69].

The development in the area of conducting experiments of the strain rate of many materials in various ranges allows for the precise determination of their characteristics and correlation with the actual conditions of their work. The commonly used experimental tests are summarized in Table 2; in particular, the respective ranges of strain rates are included [68].

## 3. SHPB Test Stand and General Principle of the Experiment

**Figure 1:**
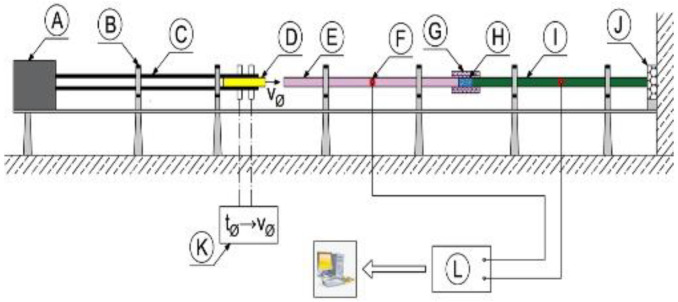
General scheme of the SHPB stand at Department of Military Engineering and Infrastructure, MUT (reprinted from Ref. [70]).

**Figure 2:**
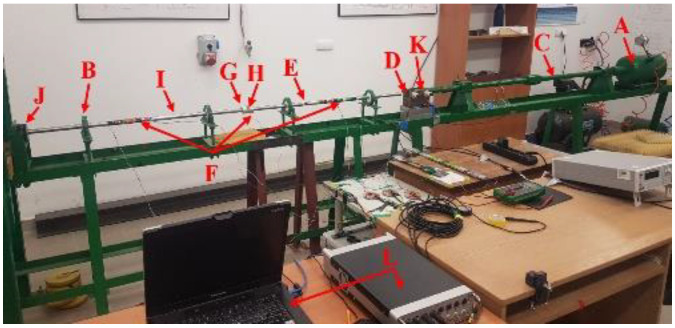
Real view of the SHPB stand at Department of Military Engineering and Infrastructure, MUT (reprinted from Ref. [70]).

The technique of the split Hopkinson pressure bar (SHPB) allows for the study and analysis of the phenomenon of co-interaction of soils and their behavior as a result of a dynamic impact with a measuring bar on the tested soil. A schematic visualization of the split Hopkinson pressure bar is shown in Figure 1 [70] and the actual view of the experimental stand in Figure 2—both drawings refer to an example of an experimental stand that is used for teaching and scientific research at the Department of Military Engineering and Infrastructure within the existing structure Faculty of Civil Engineering and Geodesy at the Military University of Technology in Warsaw.

The SHPB test stand shown in Figure 1 and Figure 2 consists of the following elements:

A—pneumatic cannon;
B—construction ensuring bar alignment;
C—barrel of loading bar projectile;
D—bar projectile;
E—initiating measuring bar;
F—strain gauge set;
G—rigid confining casing;
H—soil specimen;
I—transmitting measuring bar;
J—damper;
K—laser timekeeping system;
L—measuring device with digital memory and computer software.

**Figure 3:**
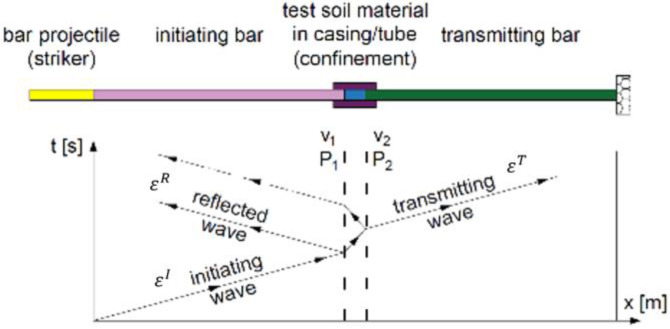
Wave diagram showing the phenomenon of propagation of elastic waves in SHPB (reprinted from Ref. [70]).

In order to facilitate the identification of individual elements of the experimental stand presented in Figure 1 and Figure 2, the letters from A to L were used. The bar projectile/striker (D) moves in the barrel of the launcher and increases its speed as a result of frontal pressure; the compressed air is rapidly released from the pneumatic gun—compressed air is supplied by the compressor (A). At the moment of leaving the barrel of the launcher, the time is measured which allows one to determine the speed of the bar projectile/striker at the moment of hitting the initiating bar. The laser light beam (K) measures the time that the bar projectile/striker has traveled the previously known measurement distance, e.g., 0.1 m. When the front of the bar projectile/striker hits the front of the initiating bar (E), an elastic wave is generated having a compressive nature (the incident wave is responsible for the formation of the initiating bar deformation $\varepsilon^{I}$), which further moves along this initiating bar toward the tested sample soil (H) placed in a casing/tube (G) dedicated to a given experiment. When the wave reaches the end of the initiating bar, the phenomenon of partial wave passage through the tested soil sample toward the transmitting bar (I) takes place (the transmitting wave is responsible for the deformation of the transmitting bar $\varepsilon^{T}$). The remaining part of the generated wave is partially reflected—it carries out the return path to the beginning of the front of the initiating bar (the reflected wave is responsible for the deformation in the initiating bar $\varepsilon^{R}$). The first part of the wave finally reaches the shock absorber (J) passing its path through the test sample and the entire length of the transmitting bar. The generated pulses received from the strain gauges are recorded and saved using a multi-channel conditioner and recorder (L). Ultimately, the data in the form of a digital signal will be transferred to dedicated computer software. The illustration of the described phenomenon is schematically shown in Figure 3 [70].

As a result of the experiment using the SHPB test stand, it is possible to calculate the strength dynamic parameters (stress σ, strain ε and strain rate $\dot{\varepsilon }$). Assumptions must be made for the further determination of the equations:

(1) $$P_{1}\left(t\right)≅P_{2}\left(t\right)$$

(2) $$\varepsilon^{I}\left(t\right)+\varepsilon^{R}\left(t\right)≅\varepsilon^{T}\left(t\right)$$

(3) $$A=A_{0}$$

where:

$P_{1}$—force at the end of the initiating bar;
$P_{2}$—force at the end of the transmitting bar;
$\varepsilon^{I}$—strain in the bar for the initiating wave;
$\varepsilon^{R}$—strain in the bar for the reflected wave;
$\varepsilon^{T}$—strain in the bar for the transmitting wave;
$A_{0}$—cross-sectional area of the sample;
A—cross-sectional area of the bars.

Equation to determine stress σ(t) as a function of time:

(4) $$\sigma \left(t\right)≅\pm E\cdot \varepsilon^{T}\left(t\right)$$

Equation to determine strain ε(t) as a function of time:

(5) $$\varepsilon \left(t\right)≅\pm \frac{2\cdot c_{0}}{L_{0}}\cdot \int_{0}^{t}\varepsilon^{R}\left(t\right)\;dt$$

Equation to determine strain rate $\dot{\varepsilon }\left(t\right)$ as a function of time:

(6) $$\dot{\varepsilon }\left(t\right)=\frac{2\cdot c_{0}}{L_{0}}\cdot \varepsilon^{R}\left(t\right)$$

where:

E—Young’s modulus of the material from which the bars are made;
$c_{0}$—elastic wave propagation velocity in the longitudinal direction of the bar;
$L_{0}$—sample length.

**Figure 4:**
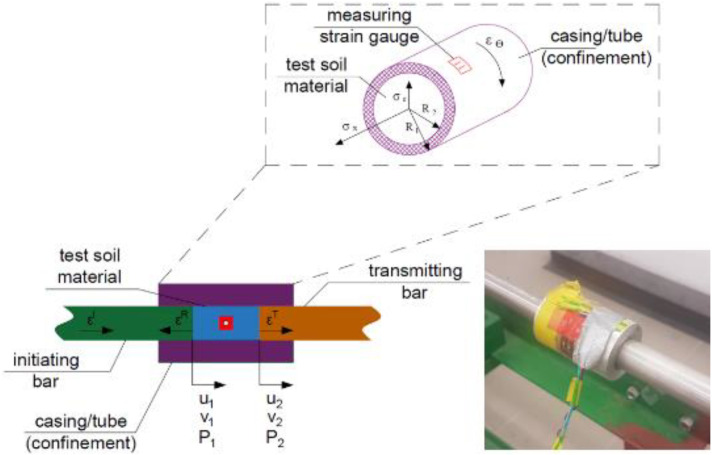
Cylindrical soil specimen in casing/tube (reprinted from Ref. [71]).

During the dynamic test using the SHPB stand, certain specific standards are applied—the test sample in the experiment has a cylindrical shape. For the duration of the experiment, the soil sample is kept in the casing/tube (Figure 4 [71])—various materials are used to make the casing/tube, adapted to the conditions of a given test, e.g., steel or duralumin. The characteristics of the material used and the ratio of the thickness and length of the casing/tube allow for uniaxial deformation of the soil sample at the moment of pressing the front initiating and transmitting bars on both sides of the tested sample fronts.

## 4. Current Trends in Soil Dynamic Research Using the Hopkinson Bar Technique

As part of this paper, a literature review was carried out in the area of dynamic tests of cohesive and non-cohesive soils using the Hopkinson bar technique ([61,66]). A compilation of the current crucial research trends in the area of soils loaded with a dynamic impact by applying the Hopkinson bar technique in 2018–2021:

- 2018

**(I)**  

**Figure 5:**
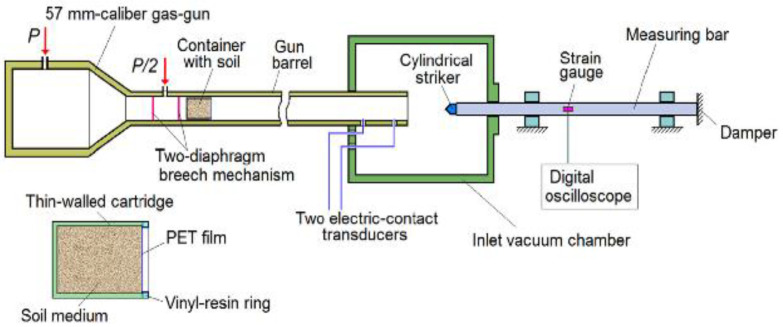
Schematic system for the experimental measurement of the forces resisting the penetration process in the reverse test (reprinted from Ref. [72]).

Test Soil Material: Dry and water-saturated sand
Authors: Bragov, A.M.; Balandin, V.V.; Igumnov, L.A.; Kotov, V.L.; Kruszka, L.; Lomunov, A.K.
Order of Cited Paper: [72]
Highlights/Abstract: The work presents and analyzes new research achievements in the field of experimental, theoretical and numerical dynamic sand interaction—experiments were performed for the cases of sand saturated with water and dry sand, which was subjected to impact and penetration with the use of cylindrical beaters (the speed range from 50 to 450 m/s). The reverse experiment technique was used—the end face of the measuring bar in the variants of the flat, hemispheric and conical heads was dynamically struck with the use of a container containing sand inside the test heads. It is possible to determine the parameters of dynamic compressibility and shear resistance of compacted water-saturated sand as a result of calculations of the maximum and quasi-stationary values of the resistance to penetration in the variant of a flat-head striker. In accordance with the conditions of the mechanics of continuous media in the axisymmetric range, a numerical analysis of the resistance parameter was performed for the penetration of impactors into the soil medium—this allowed for the determination of the parameters of the Grigoryan model. A close convergence of the results obtained between the computational and experimental analysis was confirmed. The thesis is correct that the compacted sand in the variant of complete saturation with water achieves weaker results of shear properties; nevertheless, significant values are still maintained for the dynamic impact interaction velocity. Schematic system for the experimental measurement is shown in Figure 5.

**(II)**  

**Figure 6:**
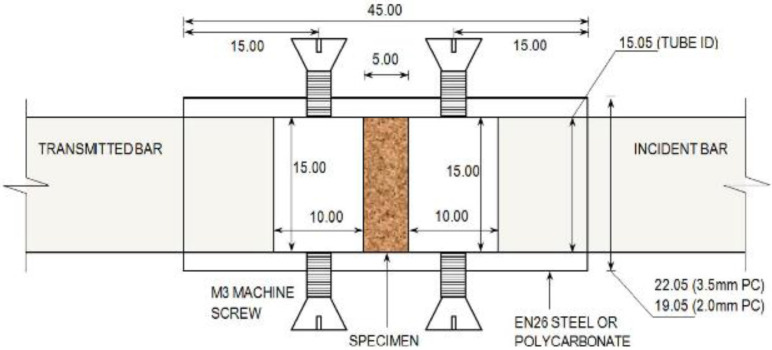
View of the soil sample inside the tube made of EN26 steel and 2.0/3.5 mm thick polycarbonate with measuring bars (reprinted from Ref. [73]). Dimensions are given in millimeters.

Test Soil Material: Water-saturated sand
Authors: Wang, S.; Shen, L.; Maggi, F.; El-Zein, A.; Nguyen, G.D.; Zheng, Y.; Zhang, H.; Chen, Z.
Order of Cited Paper: [73]
Highlights/Abstract: The sand from Stockton Beach, partially water-saturated, was tested on a split Hopkinson pressure bar to determine the compressive action at high strain rate in the soil under analysis. The essence of the research was to determine how the saturation of the sample with water at the existing initial dry sample density affects the selected parameters: initial deformation, energy absorption and grain crushing in the analyzed soil from Stockton Beach for specific experimental conditions in the form of average strain rates between 1 × 10^3^ s^−1^ and 1.3 × 10^3^ s^−1^. The samples were located inside a cured steel pipe, and the dry density was determined to be 1.46 g/cm^3^, 1.57 g/cm^3^ and 1.69 g/cm^3^ with a water saturation level of the sample from 0% to over 90%. Sand samples with a density of 1.57 g/cm^3^ during the test were also placed in polycarbonate chambers—they have a different wall thickness. After carrying out the tests, it was possible to define the conclusion that sand with partial water saturation depending on the stress–strain reaction shows stiffness increasing together with the initial density of the dry sand sample before water lock-up. This phenomenon is reversed—stiffness decreases with increasing water saturation in the sample. The tendency to increase the stiffness is generated by the stiffness of the sample closure only when enclosed in a steel tube. The phenomenon of energy absorption at the stress level present tends to increase together with a decrease in the stiffness of the tube casing (softer) and a decrease in the initial density of the dry sand sample. After the impact was performed in accordance with the test methodology, the crushed sand grains were collected for checking—a quantitative analysis of the grain crushing was performed based on Hardin’s relative fracture potential. It was observed that the occurrence of the sand grain crushing phenomenon increases with the stiffness of the tube casing and the initial dry density of the sample, while it tends to decrease linearly with increasing water saturation of the sample. The results of the tests performed are useful in the calibration and in improving the process of validation of multiphase constitutive models—it will help in determining the expected dynamic reactions in sands with partial saturation with water. View of the soil sample inside the tube is shown in Figure 6.

**(III)**  

**Figure 7:**
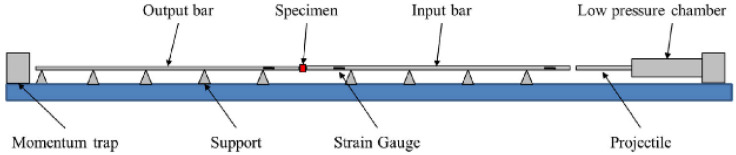
Long split Hopkinson bar setup (reprinted from Ref. [74]).

**Figure 8:**
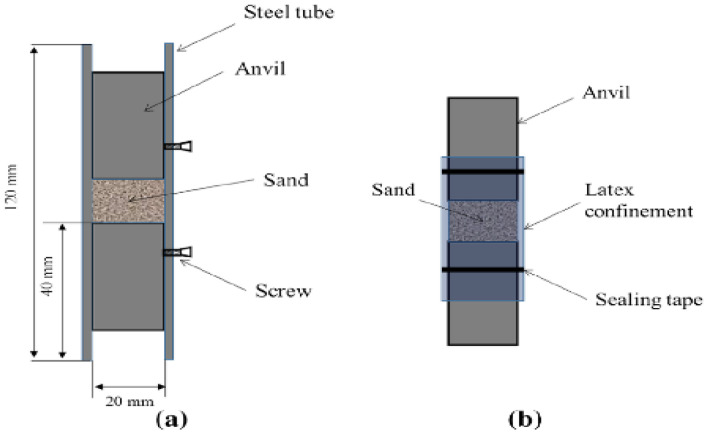
View of a casing made of (**a**) stainless steel and (**b**) latex (reprinted from Ref. [74]).

Test Soil Material: Sands - Ottawa sand, Euroquartz Siligran, Q-Rok
Authors: De Cola, F.; Pellegrino, A.; Glößner, C.; Penumadu, D.; Petrinic, N.
Order of Cited Paper: [74]
Highlights/Abstract: The experiment used a test stand in the elongated bar module—long split Hopkinson pressure bar (LSHPB). Load pulses in the range of up to 1.1 ms were generated. The study analyzed the dependence of the quantitative mechanical reaction of sand on compression at high strain rate (HSR) depending on the influence of the following factors: soil grain shape, size distribution of individual sand fractions, intergranular friction, confinement and initial compaction state. The applied test variant allowed determining the set of dynamic reactions of various types of sand in a wide strain rate range—sand samples have a compaction factor ranging from low to the highest compaction (which means the case of the lowest initial porosity coefficient). The total research analysis was based on the three types of sand used in the experiment: quasi-spherical Ottawa sand, sub-grained Euroquartz Siligran and polyhedral grain-shaped Q-Rok. For the given types of sand, the phenomena of morphology and grain size were investigated, which clearly affect the mechanical reaction in the compression variant. During the experiment, conditions enabling quasi-uniaxial strain and quasi-uniaxial stress were provided through the use of stiff (Ti64) and less rigid, deformable (latex) tube casing. An innovative achievement of this work was the determination of the effect of intergranular friction on the example of Euroquartz sand with a polymer coating. Sample preparation procedures based on representative initial consolidation states were used to maintain the realistic ranges in natural soil states from loose to dense. The conducted research is important because the results allow relating the determined parameters of the mechanical dynamic reaction in the case of HSR to the appropriate constitutive models. Long split Hopkinson bar setup is shown in Figure 7 and view of a casing made of (a) stainless steel and (b) latex is presented in Figure 8.

**(IV)**  

**Figure 9:**
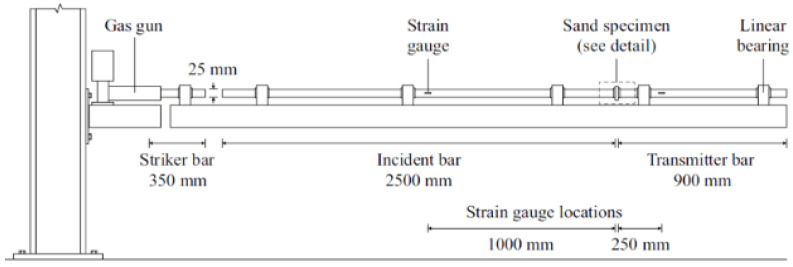
Schematic system of a modified Hopkinson split pressure bar for dynamic loading (reprinted from Ref. [75]).

**Figure 10:**
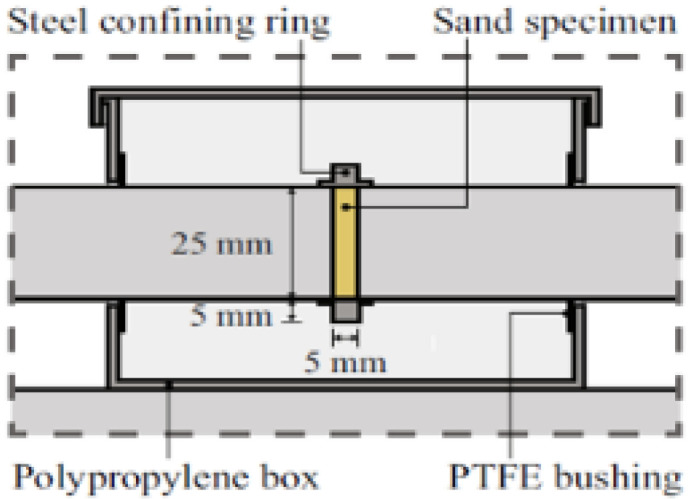
Section detail of specimen confinement (reprinted from Ref. [75]).

Test Soil Material: Quartz sand
Authors: Barr, A.D.; Clarke, S.D.; Tyas, A.; Warren, J.A.
Order of Cited Paper: [75]
Highlights/Abstract: In order to protect against the negative effects of events and various activities, e.g., explosion or fragmentation, gabion structures are used (a gabion structure is filled with one or more types of soil). The characteristic feature of these events is the strain rates and stresses at the time of their occurrence. The influence of water content in the soil in the gabion structure on the strength of the entire structure was observed—the situation of large strain rates and stresses in partially water-saturated soils is not fully known and discussed. Falling soil is a wide field for deepening further research—the behavior of this soil in the area depending on different compaction in different situations, e.g., external ballistics of a projectile requires further analyses. The paper presents tests of the compaction and compressibility of quartz sand based on the SHPB stand, depending on the sample’s water saturation level (loose soil, up to 15% water content). On the other hand, for a well-compacted sample, the water content from 0% to 7.5% decreased the characteristic stiffness of the loose soil. In terms of the water content level above 7.5%, the situation of full water-saturated soil samples occurred. Before full water-saturated (below 7.5%), additional water did not result in the stiffness of the loose soil. However, after full water-saturated (above 7.5%), additional water in the soil pores increased the stiffness of the loose soil. Schematic system of a modified Hopkinson bar is shown in Figure 9 and section detail of specimen confinement is presented in Figure 10.

- 2019

**(V)**  

**Figure 11:**
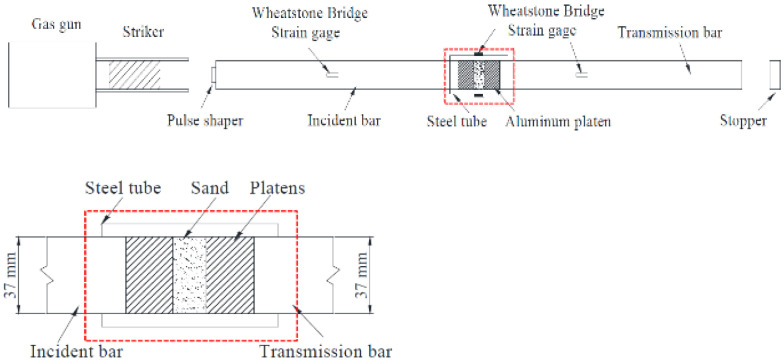
Schematic view of SHPB test with a soil specimen (reprinted from Ref. [76]).

Test Soil Material: Calcareous sand
Authors: Lv, Y.; Liu, J.; Xiong, Z.
Order of Cited Paper: [76]
Highlights/Abstract: The study focused on the analysis of calcareous sand in the area of high strain rates (HSRs)—it exhibits different characteristics compared to other soils, e.g., silica sand. The phenomenon of high strain rates occurs in many events and situations, e.g., dynamic driving of piles forming the pile foundation, mining and extraction of natural resources and utility loads caused by the movement of cars/airplanes/other vehicles. The experiment used the split Hopkinson pressure bar stand in the implementation of the calcareous sand reaction test cycle. Soil samples placed in a steel tube/casing were tested after dynamic impact, and the results were compared to the known values of the relative density and strain rate of exemplary reactions and dynamic properties of other loose soil, e.g., silica sand. In total, 6 validation tests were performed in the bar–bar version and 16 comparative tests for the dynamic properties of calcareous sand (the results were compared with silica sand). Different particle sizes, their non-annual shapes and internal composition resulted in significantly different results of the dynamic reaction of the compared soil samples—the dynamic stiffness of calcareous sand is about 10 times lower than the dynamic stiffness of silica sand. Calcareous sand is porous in nature, and in a dynamic experiment, the grains were finally crushed after the plasticity and hardening of individual grains in the soil skeleton. The destruction mechanics of a single calcareous sand particle starts with local instability and extends until it fully breaks the particle as a result of an increase in the value of the load effect. Schematic view of SHPB is shown in Figure 11.

**(VI)**  

**Figure 12:**
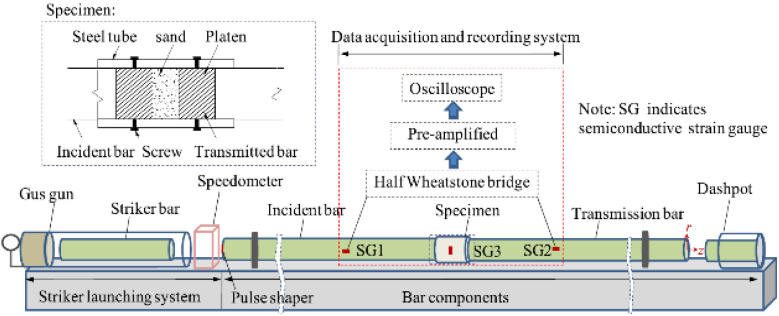
Schematic view of SHPB test stand (reprinted from Ref. [77]).

Test Soil Material: Calcareous and silica sand
Authors: Lv, Y.; Wang, Y.; Zuo, D.
Order of Cited Paper: [77]
Highlights/Abstract: The paper presents a research cycle using the split Hopkinson pressure bar stand for porous, calcareous and solid silica sands analysis. Samples of different grain sizes were used—fractions in four variants: 0.15—0.30 mm; 0.30—0.60 mm; 0.60—1.18 mm; and 1.18—2.00 mm. In the study of quartz sand, it was observed that the particle size has a significant impact on the reaction of the sample subjected to dynamic impact—the phenomenon of particle crushing appears, mainly in the last part of the strain-hardening process. Similar behavior also occurs in calcareous sand analysis; however, it continues throughout the loading process. It was measured by the fractal dimension approach that the inherent compliance trait in the crushing process of calcareous sand compared to silica sand is approximately 1.2 times stronger. Both experimental soils show opposite behaviors in terms of particle size—the larger the particle size, the more noticeable is the opposite process of changes in the void ratio and friction angle as a result of different inter-particle voids and mineral composition in these samples. Schematic view of SHPB test stand is shown in Figure 12.

**(VII)**  

**Figure 13:**
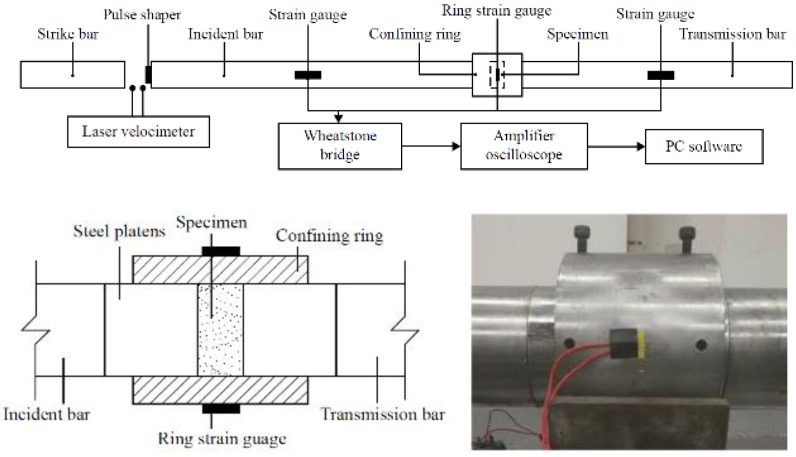
Schematic view of the SHPB test system and calcareous sand specimen (reprinted from Ref. [78]).

Test Soil Material: Calcareous sand
Authors: Wen, S.; Zhang, C.; Chang, Y.; Hu, P.
Order of Cited Paper: [78]
Highlights/Abstract: An experiment was performed on a calcareous sand sample using a split Hopkinson pressure bar with a bar length of 100 mm in order to analyze the mechanical properties for the dynamic impact case. In the study, the sample was closed in a casing/tube, and there were different test conditions: strain rates in the range of 500—800 $s^{-1}$ and pressure in the range of 0—200 MPa. An experiment was also carried out to determine the mechanical properties in the static variant in the HUT106D universal testing machine. Calcareous sand was tested under generally similar conditions as in the dynamic test—the differences were the strain rate of $2\cdot 10^{-3}\;s^{-1}$ and the pressure in the range of 0–120 MPa. Firstly, it was observed that after exceeding a certain limit value of the dynamic load, the influence of the initial pressure on the dynamic mechanical properties of the calcareous sand sample was reduced. Another conclusion was the possibility of using the Tait equation of state to present the dependence of hydrostatic pressure–volume strain in the calcareous sand sample used for the experiment in both analyzed situations—dynamic and static study. The last observation was the statement that the strain rate effect is well demonstrated by the volumetric compression degree of the analyzed calcareous sand sample. Schematic view of the SHPB test system and calcareous sand specimen are shown in Figure 13.

**(VIII)**  

**Figure 14:**
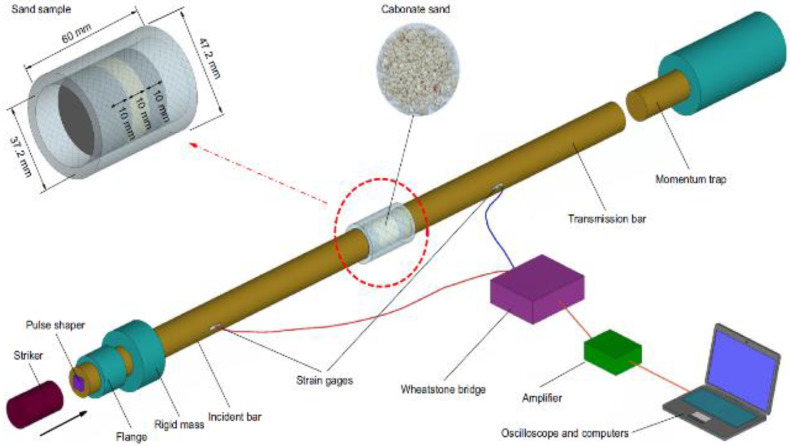
General diagram of SHPB system (reprinted from Ref. [79]).

Test Soil Material: Carbonate sand
Authors: Xiao, Y.; Yuan, Z.; Chu, J.; Liu, H.; Huang, J.; Luo, S.N.; Wang, S.; Lin, J.
Order of Cited Paper: [79]
Highlights/Abstract: The paper presents tests on carbonate sand samples as part of compression experiments: (a) with the use of the Materials Testing System for quasi-static testing and (b) with the use of split Hopkinson pressure bar for dynamic testing. In order to precisely define the particle size distributions (PSDs) in the analyzed carbonate sand samples in the pre-test and post-test situations, laser diffractometry was used. The results presented in the form of stress–strain curves prove that the carbonate sand used in the experiment reveals the influence of the strain rate effect. On the basis of the stress–strain curves, a different course of the graph was also observed for the results obtained between the tests: (a) of a quasi-static nature and (b) of a dynamic impact character. The experiment conducted under various conditions of the values of the occurring stresses and input energy showed a different range of the phenomenon of particle fracture—it was determined in detail based on the PSD in the situation before the test and after the test. The phenomenon of susceptibility to breaking of soil particles is greater in the test (a) with a quasi-static load than in the test (b) with a load resulting from a dynamic impact. For the identical value of the stress level, the breaking susceptibility of carbonate sand particles subjected to the test (b) with a load of a dynamic impact is lower than in the test (a) with a quasi-static load than in the test. Another observation was the conclusion that the fracture mechanism depends on the level of stress values—the mechanism takes the form of attrition and abrasion for low stress values, but the mechanism for high stress values is fracture. General diagram of SHPB system is shown in Figure 14.

**(IX)**  

**Figure 15:**
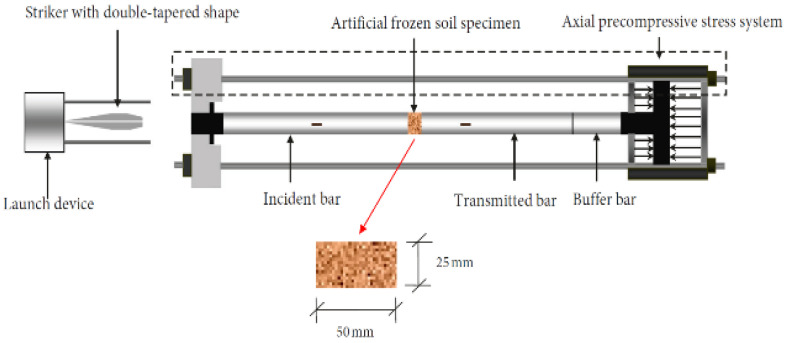
Modified SHPB test stand with frozen soil specimen - silty clay (reprinted from Ref. [80]).

Test Soil Material: Frozen Silty Clay
Authors: Ma, D.; Ma, Q.; Yao, Z.; Yuan, P.; Zhang, R.
Order of Cited Paper: [80]
Highlights/Abstract: The modified split Hopkinson pressure bar test stand allows you to analyze the dynamic behavior of a silty clay sample in an additional test module—artificial frozen sample. The dynamic hitting of a bar allows one to obtain the stress–strain dependency graph. The experiment enables the determination of several dynamic soil parameters: dynamic compressive strength, dynamic deformation modulus, energy dissipation and failure mode in the prepared sample, taking into account the axial precompressive stress ratio. During the study, the possibility of dividing the stress–strain dependency graph obtained in uniaxial and one-dimensional dynamic impact conditions into four segments was observed: (a) compaction part, (b) elastic part, (c) plastic part and (d) failure part. There was a noticeable trend of increasing values and successively decreasing them as the axial compressive stress ratio grows—the process takes place for selected dynamic parameters (e.g., dynamic compressive strength, deformation modulus for (a) compaction part, deformation modulus for (b) part elastic and absorbed energy density of the analyzed soil sample). For the axial compressive stress ratio with a value of 0.4, there is an observation that signs of the spall phenomenon are visible around the circumference of the sample, while in the central area of the sample, there is no disturbance to the soil structure. Only for the axial compressive stress ratio with a value from 0.7 to 0.9 are the signs of the shear failure process visible. There is a dependence that the higher the value of the axial compressive stress ratio, the stronger the result of the destruction process on the shear surface—for an axial compressive stress ratio of 1.0, the crush failure variant follows. Modified SHPB test stand is shown in Figure 15.

- 2020

**(X)**  

**Figure 16:**
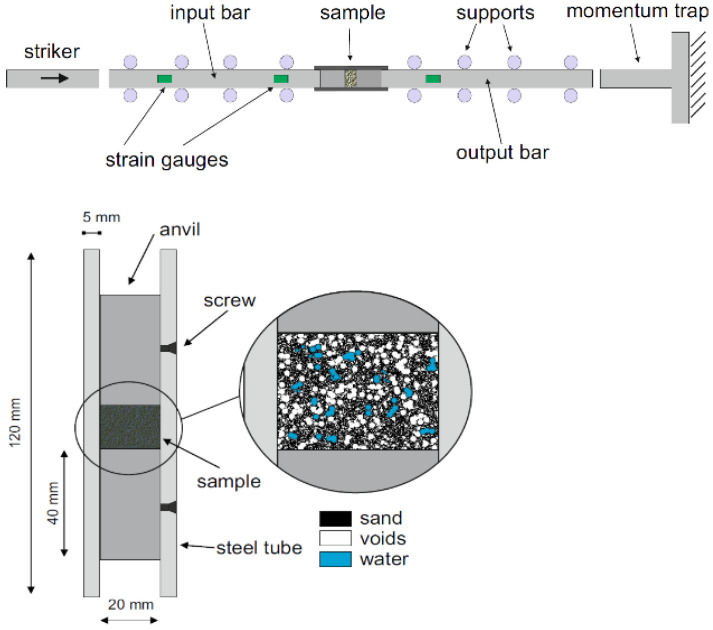
Split Hopkinson pressure bar system with soil specimen (reprinted from Ref. [81]).

Test Soil Material: Volcanic sand
Authors: Varley, L.; Rutherford, M.E.; Zhang, L.; Pellegrino, A.
Order of Cited Paper: [81]
Highlights/Abstract: Based on the split Hopkinson pressure bar test stand in the variant with an elongated bar, the soil was analyzed on the example of volcanic sand from Mount Etna in order to test the dependence of sample water moisture and the initial compaction coefficient to maintain dynamic soil subjected to a load by a projectile impact. The result of the experiment was the determination of, e.g., a graph of the dynamic dependence of stress–strains ultimately ending with a significant value of compressive strains. One of the assumptions of the experiment was to reflect the natural conditions of Mount Etna as much as possible in the research room. Volcanic sand samples with different characteristic parameters were used for the tests—samples with different percentages of water moisture and the initial porosity index. As part of the analysis, a significant influence of water (depending on the amount of its presence in the sample) on the dynamic behavior of the sample subjected to a dynamic impact with a bar was observed. The volcanic sand sample with low water content in the soil pores behaved similarly to the sample in dry conditions. It could be observed that a sample with a high percentage of water in the pores (water-saturated sample) has a significant dynamic reaction—water behaves like an incompressible material at the moment of dynamic impact, and there is a visible increase in the stiffness phenomenon at the strain of volcanic sand sample. When the sample is loaded in the quasi-static variant on a universal machine at a low strain rate, no significant influence of the water content in the sample on its behavior is observed. Additionally, an edge detection study was performed for the determination and comparative analysis of the grain fraction of the volcanic sand samples used in the experiment. Split Hopkinson pressure bar system is shown in Figure 16.

**(XI)**  

**Figure 17:**
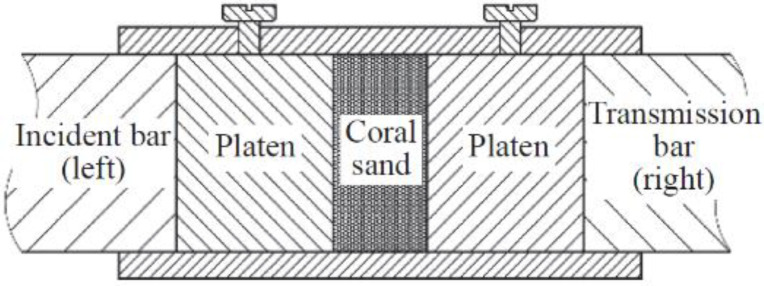
SHPB experiment section for coral sand specimen (reprinted from Ref. [82]).

Test Soil Material: Coral sand
Authors: Dong, K.; Ren, H.; Ruan, W.; Ning, H.; Guo, R.; Huang, K.
Order of Cited Paper: [82]
Highlights/Abstract: The split Hopkinson pressure bar test stand with a measuring bar diameter of 37 mm was used for experiments with two different coral sand samples in order to determine the strain rate dependence under dynamic loading of soil samples. One-dimensional stress–strain plots in various ranges of the strain rate 460 to 1300 $s^{-1}$ were determined as a result. The analysis also used the results obtained from the static machine for the strain rate $10^{-4}\;s^{-1}—$two types of coral sand showed different dynamic and static reactions during the experiment. As a conclusion, it was proposed that the susceptibility of a given type of coral sand to the strain rate is significantly dependent and related to the internal structure of grains, soil pores and the phenomenon of inter-particle friction. Additionally, proposed models supporting dynamic numerical calculations of coral sand samples as a result of dynamic impact were presented. SHPB experiment section for coral sand specimen is shown in Figure 17.

**(XII)**  

**Figure 18:**
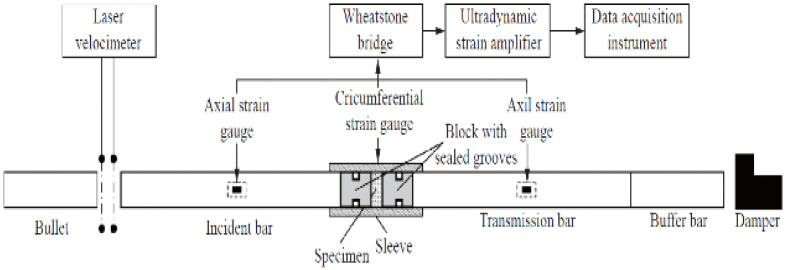
Schematic view of SHPB test stand (reprinted from Ref. [83]).

Test Soil Material: Calcareous sand
Authors: Zhao, Z.; Qiu, Y.; Zi, M.; Xing, H.; Wang, M.
Order of Cited Paper: [83]
Highlights/Abstract: As part of the experiment, the split Hopkinson pressure bar test stand was used to determine the effect of different levels of water content in calcareous sand samples under the conditions of one-dimensional state. During the research, the strain rates were obtained in the range of 209—1137 $s^{-1}$. During the experiment, it was observed that the correctness of the results significantly depends on the observance of the axial condition of the measuring bars and the performance of the calibration procedure of the characteristic parameters (e.g., sensitivity coefficient) of the set of measuring strain gauges located on the measuring bars. In the study, calcareous sand samples in the water unsaturation variant (low water moisture in the sample) were analyzed in detail, and a proposal of a dynamic behavior model was presented as a result of the stress–strain curve analysis. The impact of the limit strain value of 0.025 on the tangential modulus was observed in the tested soil type—the tangential modulus value was lower for the dry sample than for the wet sample for strain <0.025, and the tangential modulus value was greater for the dry sample than for the wet sample for strain >0.025. Schematic view of SHPB test stand is shown in Figure 18.

**(XIII)**  

**Figure 19:**
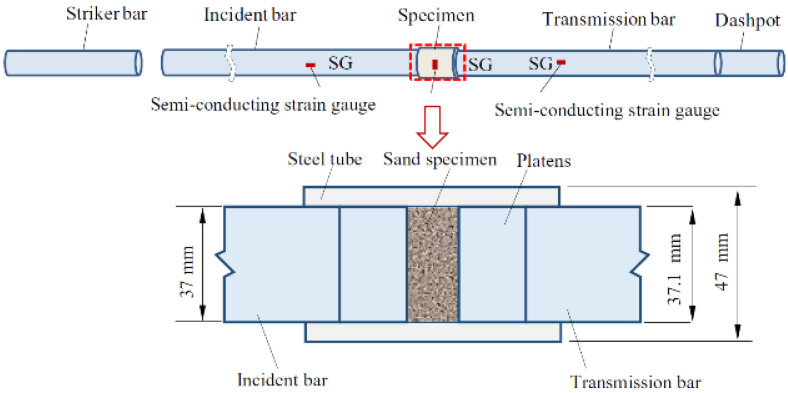
SHPB test system with sand specimen (reprinted from Ref. [84]).

Test Soil Material: Calcareous sand
Authors: Lv, Y.; Li, X.; Wang, Y.
Order of Cited Paper: [84]
Highlights/Abstract: When conducting experiments using the Hopkinson technique at high strain rates, the phenomenon of dividing into smaller parts of angular and porous soil grains takes place—in this study, calcareous sand was analyzed. In addition to observing this process, it is also worth quantifying how many grains it concerns. A series of tests were performed and analyzed using the split Hopkinson pressure bar test stand to discuss how various variable conditions in the test (impact energies, relative densities, water moisture content and particle gradations) affect the phenomenon of calcareous sand grain breakage. It was observed that the process of grinding and reordering the soil grain structure continues during the entire dynamic loading process—it results from the analysis of the stress–strain curve of the tested soil sample in an almost linear form. The parameter relating to the particle breakage depends in an exponential way on the value of the impact energy of the bar projectile. In the beginning, for a soil sample with a low water content, a decrease in the particle breakage phenomenon is observed; a further increase in the soil moisture level causes an increase in the occurrence of particle breakage during the observation. The result of the study is also the conclusion that (a) for calcareous sand samples when saturated with water, larger-diameter particles are damaged more, and those with small diameter are less damaged, and (b) for calcareous sand samples with low water saturation or dry, particles with a small diameter are more damaged in the process, while those of a larger diameter are less damaged. SHPB test system with sand specimen is shown in Figure 19.

**(XIV)**  

**Figure 20:**
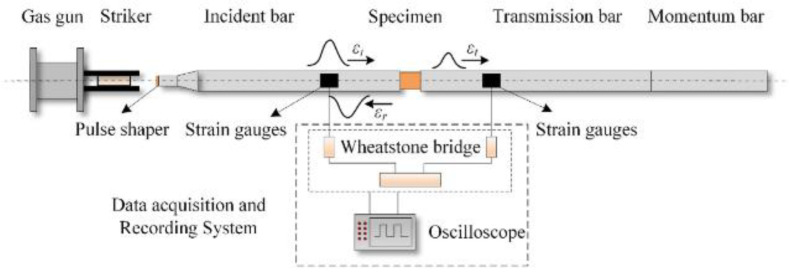
Split Hopkinson pressure bar system (reprinted from Ref. [85]).

Test Soil Material: Frozen soil
Authors: Zhang, F.; Zhu, Z.; Fu, T.; Jia, J.
Order of Cited Paper: [85]
Highlights/Abstract: The paper deals with the subject of dynamic mechanical properties as a result of tests using the split Hopkinson pressure bar test stand under HSR conditions for an experiment with a soil sample in the variant of a reduced temperature (frozen soil). The study focused on the process of changing the wave impedance value for the case of a frozen soil sample. A concordance was observed between the viscoelastic theory record and the wave impedance increase experiment when the sample was prepared by freezing. The indicated phenomenon results from the relaxation of water that has not been completely frozen—it results in an increase in the maximum stress values as a result of a dynamic bar impact on a frozen soil sample. The work used the effective medium theory by taking into account the macroscopic parameter (velocity of the propagated wave) and the mesoscopic parameter (random or vertical microcrack mesh density), assuming the variable damage as the longitudinal wave propagation speed. The Zhu–Wang–Tang model was included in the dynamic analysis, which, for the Maxwell element (in the form of low-frequency parameters) in the assumed conditions of a frozen soil sample, did not finally fulfill the properties of a simple spring—this correlates with the observed macroscopic properties of the soil sample in the freezing variant. The result of the work was the development of a dynamic constitutive model for the analyzed soil type in the frozen sample variant (empirical and experimental results are comparable). Split Hopkinson pressure bar system is shown in Figure 20.

- 2021

**(XV)**  

**Figure 21:**
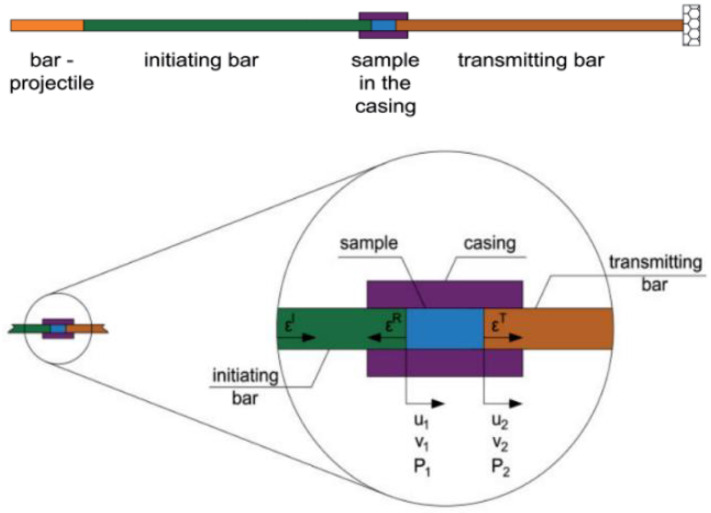
Detailed view with the sample in contact with the fronts of both measuring bars (reprinted from Ref. [71]).

Test Soil Material: Silty sand
Authors: Chmielewski, R.; Kruszka, L.; Rekucki, R.; Sobczyk, K.
Order of Cited Paper: [71]
Highlights/Abstract: Silty sand was subjected to a dynamic impact bar on a split Hopkinson pressure bar test stand. The aim of the experiment was to obtain the dependence graphs of stress–stain curves and strength dynamic parameters under changing conditions: (a) different strain rate values and (b) different values of water moisture in the sample. In the study, the possibility of sideways deformation of the sample was limited by sufficiently high tube/casing stiffness, obtaining oedometric conditions. A sieve analysis of the soil sample was performed, and the percentage content of individual fractions was determined—the largest percentage is the fine fraction. The use of the analyzed silty sand sample in a rigid casing/tube made of duralumin material allows the assumption of uniaxial deformation conditions in the experiment. The digital data recording was transferred to the computer through the use of a set of strain gauges on each of the two bars that were elements of the test stand (initiating bar and transmitting bar). An additional set of strain gauges was also glued onto the rigid casing/tube for peripheral results. During the experiment, the nature and values of the curves of three types of waves were determined—incident, reflected and transmitted waves created as a result of a dynamic impact with a bar. Soil samples of dominant sandy fractions and silts showed different density values in the standard optimal humidity test (Proctor test) than in the case of dynamic compaction under HSR (high strain rate) conditions. The cause of the phenomenon is a change in the type of soil particles through the fracture mechanism of the soil structure as a result of a large increase in the damage energy balance from bar impact. A unique element of the work was that it carried out and showed the results through a particle size distribution test curve for two different types of silty sand samples. Detailed view with the sample in contact with the fronts of both measuring bars is shown in Figure 21.

**(XVI)**  

**Figure 22:**
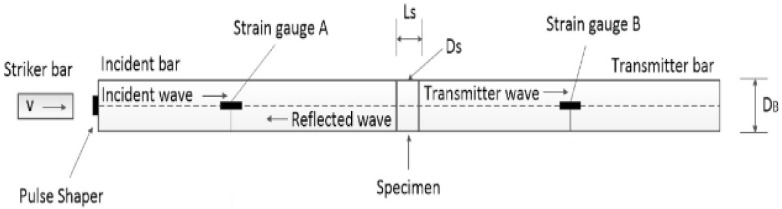
General view of SHPB test system (reprinted from Ref. [86]).

Test Soil Material: Frozen soil
Authors: Zhu, Z.; Fu, T.; Zhou, Z.; Cao, C.
Order of Cited Paper: [86]
Highlights/Abstract: An important area in the construction industry is the influence of temperature (e.g., the situation between summer and winter) on dynamic reactions resulting from the influence of dynamic load. The study analyzed the dynamic mechanical properties of the soil sample in the freezing variant—soil samples with a water content of 20% and freezing temperatures of different values were tested on a split Hopkinson pressure bar test stand, where the soil was impacted by a dynamic bar projectile. The research on the dynamic reaction focused on two areas: (a) increase in the temperature of the soil sample and (b) mechanism of the failure process. During the development of the experiment results, the Ottosen model was used, including the assumptions and conditions of thermal activation theory, and consequently, the rate damage equation was determined. The final conclusion was the recommendation that an improved non-linear mode should be used for the dynamic and mechanical analysis of soil. General view of SHPB test system is shown in Figure 22.

**(XVII)**  

**Figure 23:**
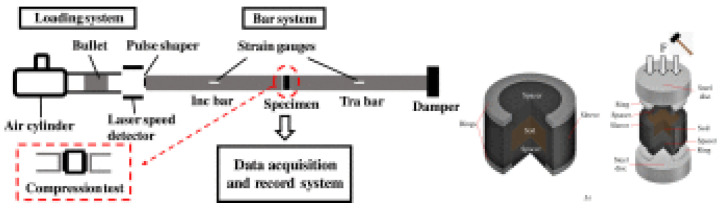
Schematic view of SHPB test stand and a specimen (reprinted from Ref. [87]).

Test Soil Material: Sandy soil
Authors: Li, T.; Li, G.; Ding, Y.; Kong, T.; Liu, J.; Zhang, G.; Zhang, N.
Order of Cited Paper: [87]
Highlights/Abstract: The paper analyzes sandy soil samples—it is a type of soil that often occurs in nature as a type of geotechnical material. Based on the tests on the SHPB test stand, the variable test conditions were analyzed, such as soil sample moisture and soil sample compaction value on the response of dynamic mechanical properties as a result of the dynamic load of the bar projectile. It was observed that dry or low-water sandy soil samples for the strain rate values between 500 and 2200 s^−1^ were characterized by an increase in the dynamic reaction. The dynamic response can be enhanced by changing the sample conditions—higher water content in the soil (sample hydration) and a higher degree of compaction (additionally densifying the sample). It was shown that the results of the experiment for the soil with comparable values of the compaction degree of 97.5% and 100% had no significant differences; only significant deviations in the results were observed when comparing these values with 95%. It was also observed that the degree of humidity at the level of 15% causes an increase of about two times the dynamic mechanical properties in comparison with the soil samples with low water content. Another important factor is the type of the sleeve material—the sandy soil sample shows a different response to the dynamic impact of the bar projectile between the situation with the steel sleeve and the aluminum sleeve. The phenomenon is a result of the conditions for the triaxial stress state and the level of the Poisson’s ratio value—assuming the same strain rate value, the sandy soil sample in the steel sleeve will show fewer dynamic properties than the sandy soil sample in the aluminum sleeve. Schematic view of SHPB test stand and a specimen are shown in Figure 23.

**(XVIII)**  

**Figure 24:**
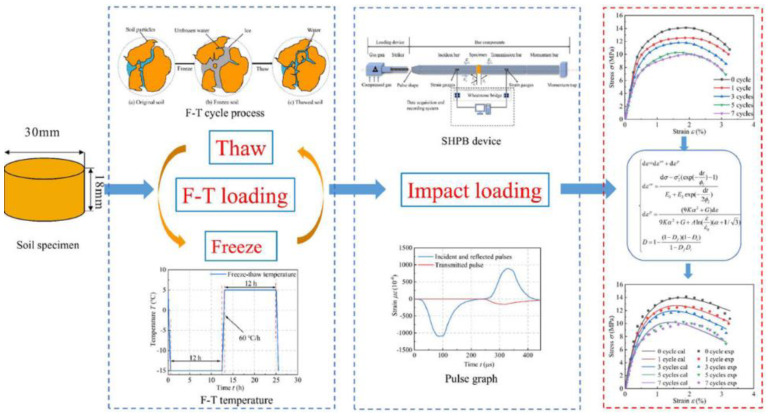
Research procedure of the conducted SHPB experiment (reprinted from Ref. [88]).

Test Soil Material: Frozen soil
Authors: Li, B.; Zhu, Z.; Ning, J.; Li, T.; Zhou, Z.
Order of Cited Paper: [88]
Highlights/Abstract: The paper focuses on the analysis of the effect of the complex freezing–thawing process for the values of dynamic mechanical properties of soil samples subjected to dynamic loading from a bar projectile/striker impact. The experiment used a split Hopkinson pressure bar test stand with a soil sample and a variable in the form of the number of freeze–thaw cycles performed and different temperature levels of these cycles. A significant influence of the number of performed freezing–thawing complex processes on the dynamic reaction of the soil sample was observed. The following results were observed on the basis of the test cycle: (a) the greater the number of freeze–thaw cycles, the lower the maximum stress values in the soil sample; (b) the limit number of freeze–thaw cycles for a balanced state of stress values in the sample is in range from 3 to 7; and (c) the lower the sample temperature in the freeze–thaw cycle, the lower the maximum stress values achieved. The study combined the discretized Zhu–Wang–Tang model and the theory of plasticity according to the condition of the plastic Drucker–Prager criterion. As a result of this integration, a constitutive model was developed taking into account the viscoelastic-plastic character area of the soil model dynamically loaded with a bar impact in the range of variable temperature conditions of the freeze–thaw cycle process. Finally, the results of the SHPB test were confirmed with the constitutive model developed. Research procedure of the conducted SHPB experiment is shown in Figure 24.

**(XIX)**  

**Figure 25:**
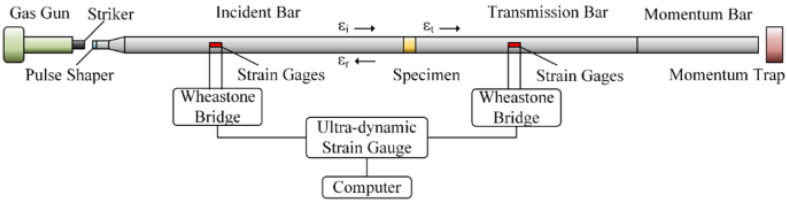
General view of SHPB system (reprinted from Ref. [89]).

Test Soil Material: Frozen soil
Authors: Jia, J.; Tang, H.; Chen, H.
Order of Cited Paper: [89]
Highlights/Abstract: The paper presents the results of the experiment carried out on the basis of the split Hopkinson pressure bar test stand for various strain rates with the use of a soil sample in a variable temperature variant—the sample was frozen. The result of the research is a graphical plot of the strees–strain variability in the analyzed soil sample dynamically loaded with bar projectile. It was observed that during the test of a frozen soil sample with a dynamic load caused by a bar impact, the shear fracture phenomenon appears near the elastic limit. As a result, the ability of water to maintain bearing capacity (in frozen form—ice) in the pores of the soil is significantly lost. The role of stress transmission is mainly performed by the soil skeleton. In addition, it was established that there is a visible relationship between the strain rate values and the levels of freezing temperature used for the experiment, affecting the results of the study in the form of dynamic mechanical properties in soil samples. It was observed that (a) soil parameters’ secant modulus, elastic modulus and strength increase their values for the case of an increase in the loading strain rate value; (b) soil parameters secant modulus, elastic modulus and strength increase their values when the freezing temperature of a soil sample is lowered; and (c) as a result of a dynamic impact of a bar projectile on the sample: strain of the soil sample, damage propagation and the process of phase transformation of melting from ice into water by providing additional energy. General view of SHPB system is shown in Figure 25.

## 5. Discussion

This paper focuses mainly on tests with measuring bars in the compression module. However, this method does not allow us to find out the full characteristics of the tested sample. In tests using the Hopkinson technique, various modules of the Hopkinson bar can be used to determine the dynamic mechanical properties of the tested soil samples (or other samples, e.g., steel, concrete, ceramics)—the appropriate module should be selected depending on the type of sample of the tested material and the results sought:

- Compression;
- Tension;
- Shear;
- Crack resistance;
- Dynamic friction;
- Hardness (penetration);
- Bauschinger effect;
- Brazilian test (splitting test).

The main focus of this review in Section 4 is the compilation of selected experiments using the compression module through dynamic testing using the split Hopkinson pressure bar in the 1D configuration. The 1D configuration is more often used and more common in various universities/research centers around the world than the 3D configuration—it can be noticed by comparing the highlights/abstracts from Section 4. The limitations of using the Hopkinson technique are shown in Table 1—the key is the strain rate expected to be obtained by the researcher. For Hopkinson’s technique, the strain rate range is between the values $10^{2}–10^{4}$ $s^{-1}$. In order to determine the mechanical properties of the tested soil sample in other ranges of the strain rate, a different test stand should be used:

- For the range ultra-high strain rate $10^{4}–10^{8}$ $s^{-1}$—Taylor impact/plate impact;
- For the range medium strain rate $10^{0}–10^{0}$ $s^{-1}$—hydraulic devices;
- For the range quasi-static and creep and stress relaxation strain rate $10^{0}–10^{-8}$ $s^{-1}$—conventional cross-head devices.

Experimental research taking into account the ranges of the strain rate is summarized in Table 1 and Table 2.

Analyzing critically the observations and achievements of the publications presented in Section 4, attention should be paid mainly to:

(a) The type of soil used in the tested samples

There is a greater tendency to conduct research in non-cohesive types of soil (mainly sandy) than in cohesive soils:

- Quartz sand—a particularly good analysis was performed in [75], where the conclusion is valuable that before full water-saturated (below 7.5%), additional water did not result in the stiffness of the loose soil. However, after full water-saturated (above 7.5%), additional water in the soil pores increased the stiffness of the loose soil;
- Calcareous sand—a very good analysis was performed in [78], where a valuable note is that after exceeding a certain limit value of the dynamic load, the influence of the initial pressure on the dynamic mechanical properties of the calcareous sand sample was reduced;
- Carbonate sand—a particularly good analysis was performed in [79], where the important conclusion is that the fracture mechanism depends on the level of stress values—the mechanism takes the form of attrition and abrasion for low stress values, but the mechanism for high stress values is fracture;
- Volcanic sand—a particularly valuable analysis was performed in [81], with a conclusion that a sample with a high percentage of water in the pores (water-saturated sample) has a significant dynamic reaction—water behaves like an incompressible material at the moment of dynamic impact, and there is a visible increase in the stiffness phenomenon at the strain of volcanic sand sample;
- Coral sand—a particularly good analysis was performed in [82], where an important observation is that the susceptibility of a given type of coral sand to the strain rate is significantly dependent and related to the internal structure of grains, soil pores and the phenomenon of inter-particle friction;
- Silty sand—a particularly valuable analysis was performed in [71], where a unique element of the work was that it carried out and showed the results through a particle size distribution test curve for two different types of silty sand samples subjected to the same bar-projectile impact.

Particularly valuable are comparative analyses of dynamic research of two or more types of soil:

- In [74], the soil quasi-spherical Ottawa sand, sub-grained Euroquartz Siligran and polyhedral grain-shaped Q-Rok research was conducted, where an innovative achievement of this work is the determination of the effect of intergranular friction on the example of Euroquartz sand with a polymer coating;
- Dynamic calcareous and silica sand studies were performed in [77], where the important conclusion is that experimental soils show opposite behavior in terms of particle size—the larger the particle size, the more noticeable is the opposite process of changes in the void ratio and friction angle as a result of different inter-particle voids and mineral composition in these samples.

On the other hand, a smaller tendency for experiments is visible for cohesive soils; nevertheless, the results are equally important for tests in non-cohesive soils. In [80], dynamic studies of silty clay were performed, where it is worth noting the conclusion that there is a dependence that the higher the value of the axial compressive stress ratio, the stronger the result of the destruction process on the shear surface—for an axial compressive stress ratio of 1.0, the crush failure variant follows.

(b) The level of water content in the tested soil sample

Many experiments cited in this paper showed the influence of water content in the sample on the dynamic mechanical properties of the soil. In all analyzed studies, it was observed that such a dependence of the influence of water in the sample on the results of dynamic soil behavior is true. In the vast majority of studies, the situations between the dry state of the sample and the state of full water saturation in the sample are compared [72]. Experiments with soil samples with several levels of water content are also valuable—in the work of [71], samples with four different levels of water content were tested, where these values were related to the optimal humidity. It can be seen that the level of optimal humidity is a clear border of the different dynamic responses of the non-cohesive soil sample. It is valuable to compare the dynamic reaction of the soil for extreme situations—a dry sample and a sample fully saturated with water. In [72], the key conclusion is that the compacted sand in the variant of complete saturation with water achieves weaker results of shear properties; nevertheless, significant values are still maintained for the dynamic impact interaction velocity. Additionally, in [73], it is important to conclude that sand with partial water saturation depending on the stress–strain reaction shows stiffness increasing together with the initial density of the dry sand sample before water lock-up and stiffness decreases with increasing water saturation in the sample.

(c) Non-standard temperature conditions of the soil sample

As a result of the ongoing climate change, it is crucial to understand soil characteristics, including soil dynamic properties, in various temperature ranges inside the soil sample. The main focus of the research area is focused on experiments with a frozen soil sample. A valuable observation was obtained in [89] that during the test of a frozen soil sample with a dynamic load caused by a bar impact, the shear fracture phenomenon appears near the elastic limit. As a result, the ability of water to maintain bearing capacity (in frozen form—ice) in the pores of the soil is significantly lost. In the area of research with non-standard soil temperature, experiments as part of the complex freezing–thawing process are of particular importance. In [88], unique tests of subjecting a soil sample to dynamic action after carrying out freeze–thaw cycles were performed. The important conclusions from the experiment were that the greater the number of freeze–thaw cycles, the lower the maximum stress values in the soil sample, and the lower the sample temperature in the freeze–thaw cycle, the lower the maximum stress values achieved.

Another research path that allows for dynamic soil experiments is the possibility of using the Hopkinson technique in a 3D configuration—triaxial Hopkinson bar. Publication [90] presents the original version of the research using the Hopkinson technique—the three-dimensional SHPB test stand. The difference of the SHPB test stand between the 1D and 3D configuration is that 1D is one-dimensional dynamic impact and 3D is limited-pressure dynamic impact.

**Figure 26:**
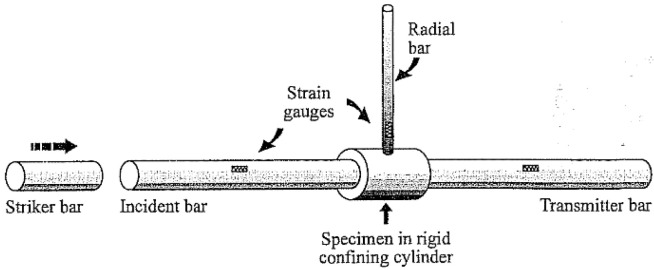
Diagram of the three-dimensional SHPB test stand (reprinted from Ref. [90]).

Using a rigid confining casing allows one to achieve a situation where the radial strain values are zero. In this 3D configuration, there is an additional bar—radial bar, which contacts directly and perpendicularly with the surface of rigid confining casing in accordance with Figure 26.

**Figure 27:**
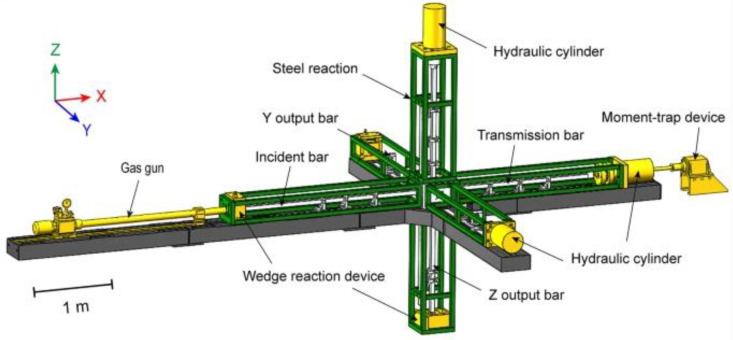
Schematic diagram of the Hopkinson bar triaxial test stand (reprinted from Ref. [93]).

**Figure 28:**
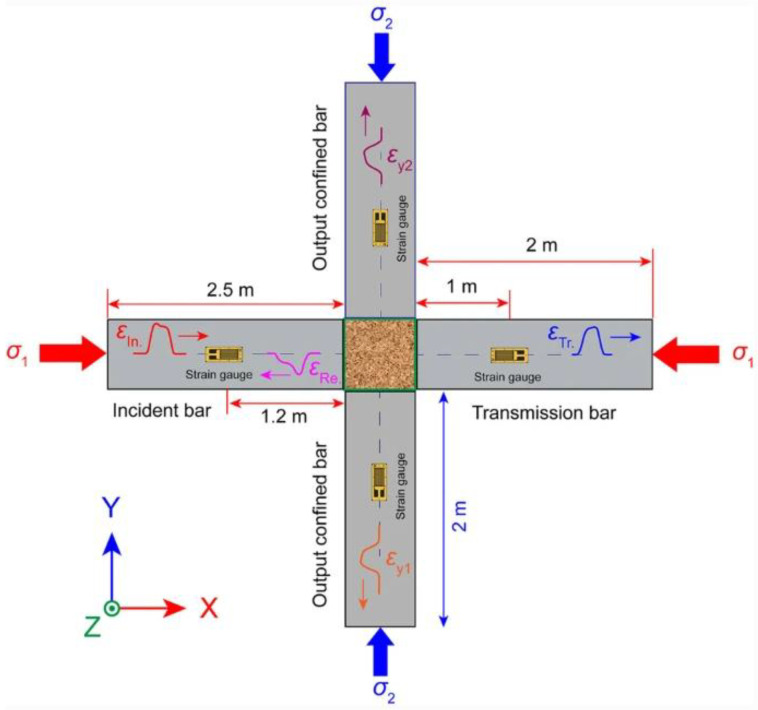
Schematic wave propagation process in 3D configuration (reprinted from Ref. [93]).

The equipment in the 3D configuration consists of: two axial measuring bars (initiating bar and transmitting bar), which allow one to measure the forces and displacements of the measuring bars axially in the X direction, and an additional one in the 3D radial bar configuration in the Y direction, which allows one to perform a radial stress measurement during the experiment. The main advantage of the 3D configuration was indicated—it enables the measurement and obtaining a comprehensive three-dimensional soil reaction to the dynamic impact. The dynamic response is more extensive compared to the classic 1D SHPB configuration. Publications [91,92] show another advantage of the 3D configuration as the widespread use of this variant of the experiment to measure the shear strength of a soil sample. It is possible to determine the dynamic characteristics of the soil response to HSR. An example of research on the dynamic behavior of sand samples (in conditions without drainage) was used to determine the influence of strain rate in the stress–strain relation. The main result shows an additional advantage of this configuration and indicates that the dynamic response of the soil sample as a stress–strain relation is more sensitive to the adopted pressure value in the test and not very sensitive to the level of loading rates. The final results in the 3D configuration may be the basis for the development of a constitutive model of this soil as a result of taking into account the triaxial stress state. Publication [93] shows a diagram of a triaxial Hopkinson bar test stand (Figure 27). The equipment is a research system that allows one to perform dynamic experiments in the uniaxial, biaxial and triaxial variants. It is worth noting that the bars are used as three pairs of test bars in three perpendicular directions X, Y and Z. In the X direction, a set of measuring bars (initiating and transmitting) in the 1D configuration is normally located. In addition, there are bars in the Y and Z directions—at their ends, there are load cylinders that limit the pressure in the bars. Figure 28 presents a schematic process of wave propagation: (a) a standard for 1D configuration is provided: $\varepsilon_{I}$—initiating wave, $\varepsilon_{R}$—reflected wave, $\varepsilon_{T}$—transmitting wave; and (b) additional for 3D configuration: $\varepsilon_{y1}$ and $\varepsilon_{y2}$—confining wave.

The results of the experiments in the triaxial Hopkinson bar configuration expand the research scope of understanding the full and complex dynamic characteristics of the tested soil sample, taking into account the triaxial state of stress in the X, Y and Z directions.

In the work based on the Hopkinson technique, an analysis of the dynamic behavior of soils, in particular sands, was carried out as a phenomenon of a “continuum” nature. The actual real behavior of soil (granular material) subjected to dynamic influence is different—the nature of the phenomenon is related to the particle interaction process in the moment of contact. The results of the multi-scale analysis of dynamic behavior of sands are published, which include analyses assessing the extent to which particle scale characteristics affect the dynamic interactions of the soil [94,95]. The important features that affect the dynamic response of the soil include: contact behavior of sand grains, morphology and origin.

## 6. Conclusions

The paper presents a literature review of crucial experimental tests of strength properties of soils (both cohesive and non-cohesive) in the area of high strain rates (HSRs) for the purpose of proper selection of structural design solutions for buildings, protective elements and critical infrastructure. In these dynamic physics experiments, the split Hopkinson pressure bar test stand is applicable.

The use of Hopkinson’s technique in experiments to determine the dynamic properties of various materials, including cohesive and non-cohesive soils, is limited by the limited range of the strain rate. In order to determine the full characteristics of a given material in different strain rate ranges, tests should be performed based on other measuring devices, e.g., conventional cross-head devices for the quasi-static range. Depending on the dynamic mechanical properties tested and the expected results, it is necessary to select an appropriate test module for measuring bars and sample sizes, e.g., compression, tension and shear. The use of different test-type modules allows for more extended results.

The paper shows the SHPB test stand in 1D and 3D configuration. Sample diagrams are provided which include an additional radial bar in direct contact with the rigid confining casing and perpendicular to the other measuring bars (initiating and transmitting). The main advantage of using the 3D configuration was indicated—the possibility to understand the full triaxial dynamic response of the soil. The analyzed publications confirm the conclusion of the results with the use of the SHPB in the 3D configuration—in the example of sand, a very weak correlation of the initial density parameter value and the strain rate value in the stress–strain relationship was shown, while a strong correlation of the pressure level with the stress–strain relationship was demonstrated.

The paper presents a review of research patterns of physical experiments from 2018-2021. The current research trends in the area of soils loaded with dynamic interaction as a result of the application of the Hopkinson bar technique are presented. These studies show that the dynamic response of the soil depends on various factors: density, cohesion, moisture content and the grain structure of the soil specimen. Soils are sensitive to the strain rate, e.g., their initial modulus is greater if the specimens are dynamically loaded with a constant value of the strain rate and higher value of the initial density comparing these values to specimens subjected to static loading and dynamic loading with a lower initial density. Moreover, the thickened and saturated soil swells at a high strain rate. “Weaker” soil response to dynamic loads is observed at lower strains with the state of incomplete water saturation, while the “stiff” response occurs at higher strains related to the state of full water saturation (noticeable influence of the water saturation level in the soil specimen). There is also a noticeable increase in the number of SHPB experiments performed in both 1D and 3D versions under modified conditions (frozen/heated soil specimen, different degree of water saturation of the soil specimen) in a wide range of strain rates, which is a large field for further research. In order to determine the constitutive model of the tested soil, it is necessary to extend the research with other equipment in order to be able to determine all the required coefficients and frequencies of the adopted model.

Based on the conducted review of selected experiments with the use of the SHPB test stand in order to determine the dynamic mechanical properties of soil samples, it is possible to notice development trends in this area. It is evident in the 2018–2021 period under investigation that SHPB experiments are carried out in various universities/research centers around the world on many types of cohesive and non-cohesive soil samples—there is a greater research tendency for non-cohesive soil, in particular: quartz sand, calcareous sand, carbonate sand, volcanic sand, coral sand and silty sand. The most common variable in the research is the change in the water content in the soil sample—the research mainly analyzed the dynamic behavior of the soil in extreme moisture cases: dry soil and soil fully saturated with water. Less frequently, the investigators analyzed the dynamic behavior of the soil for several step changes in the water content levels in the sample (together with the determination of the optimal moisture value).

Based on the conducted review of experiments, the probable prospect of research development can be defined as:

- Further research work based on the SHPB test stand in the 1D configuration for various types of cohesive and non-cohesive soils (in particular for less common types of soil, which are insufficiently tested so far);
- Increasing the number of SHPB test stands in 3D configuration at universities/scientific institutions in the world in order to understand the full triaxial behavior of dynamic soil;
- Due to the advancing climate change in the world, it will be necessary to further understand the dynamic properties of various soils under non-standard temperature conditions and conduct research using the Hopkinson technique for heated and frozen soil samples (in particular, experiments are valuable where the sample is tested in freeze–thaw cycles).

## Figures and Tables

**Table 1:** List of ranges of strain rate and experimental techniques.

$\mathrm{Strain}\;\mathrm{Rate}\;(s^{-1})$	Experimental Techniques				
$10^{8}\;$	Inertia important	Shock/Ultra-high	Plate impact		
$10^{6}\;$			Taylor impact		
$10^{4}\;$	Inertia negligible	High rate	Hopkinson bar		
$10^{2}\;$			Hydraulic devices	Hard impact (missiles, rock falls)	Earthquake and induced shocks
$10^{0}\;$		Medium rate/ Intermediate			
$10^{-2}\;$		Quasi-static	Conventional cross-head devices	Plane crash	
$10^{-4}\;$		Creep and stress relaxation		Vehicle impact	
$10^{-6}\;$				Static load (consolidation, rheology)	
$10^{-8}\;$					

**Table 2:** Experimental research taking into account the ranges of the strain rate.

$\mathrm{Strain}\;\mathrm{Rate}\;(s^{-1})$	Experimental Techniques
Compression tests	
Below $10^{-1}$	Conventional load frames
$10^{-1}–10^{2}$	Special servo-hydraulic frames
$10^{-1}–0.5\cdot 10^{3}$	Cam plastometer and drop test
$10^{1}–10^{4}$	Split Hopkinson pressure bar
$10^{3}–10^{5}$	Taylor impact test
Above $10^{5}$	Single- and two-stage gas gun
Tension test	
Below $10^{-1}$	Conventional load frames
$10^{-1}–10^{2}$	Special servo-hydraulic frames
$10^{2}–10^{3}$	Split Hopkinson pressure bar (in tension version)
$10^{4}$	Expanding ring
Above $10^{5}$	Flyer plate
Shear and multiaxial tests	
Below $10^{-1}$	Conventional shear tests
$10^{-1}–10^{2}$	Special servo-hydraulic frames
$10^{1}–10^{3}$	Torsional impact
$10^{2}–10^{4}$	Split Hopkinson pressure bar (in torsion version)
$10^{3}–10^{4}$	Double-notch shear and punch
$10^{4}–10^{7}$	Pressure-shear plate impact

## Data Availability

Data available in a publicly accessible repository.

## References

[1] (2021). Annual Threat Assessment of the US Intelligence Community.

[2] (2021). The World Climate and Security Report.

[3] (2021). The Global Risks Report.

[4] Falta J., Zlámal P., Adorna M. (2018). Instrumentation of Split Hopkinson Pressure Bar for testing of cellular metallic materials. Acta Polytech..

[5] Adorna M., Zlámal P., Fíla T., Falta J., Felten M., Fries M., Jung A. (2018). Testing of hybrid nickel-polyurethane foams at high strain-rates using Hopkinson bar and digital image correlation. Acta Polytech..

[6] Markovsky P.E., Janiszewski J., Bondarchuk V.I., Stasyuk O.O., Savvakin D.G., Skoryk M.A., Cieplak K., Dziewit P., Prikhodko S.V. (2020). Effect of Strain Rate on Microstructure Evolution and Mechanical Behavior of Titanium-Based Materials. Metals.

[7] Panowicz R., Janiszewski J., Kochanowski K. (2019). Effects of Sample Geometry Imperfections on the Results of Split Hopkinson Pressure Bar Experiments. Exp. Tech..

[8] Moćko W., Kostrzewski C., Brodecki A. (2018). Influence of anisotropy on the viscoplastic properties of a hot rolled ti6al4v titanium alloy. Arch. Metall. Mater..

[9] Bragov A.M., Konstantinov A., Lomuniv A.K., Stolyarov V.V., Kuznetsov A.V., Smakovsky M.S., Savenkov G.G. (2021). Features of Dynamic Deformation and Failure of Aluminum Bronze Processed by Laser Surface Treatment. J. Dyn. Behav. Mater..

[10] Tang W., Kang S., Liu Z. (2021). Dynamic Compression Deformation and Constitutive Model of Extruded Mg–1Zn–2Y Bar. Trans. Indian Inst. Met..

[11] Chen G., Ge J., Lu L., Liu J., Ren C. (2021). Mechanism of ultra-high-speed cutting of Ti-6Al-4V alloy considering time-dependent microstructure and mechanical behaviors. Int. J. Adv. Manuf. Technol..

[12] Golasiński K.M., Janiszewski J., Sienkiewicz J., Płociński T., Zubko M., Świec P., Pieczyńska E. (2021). Quasi-Static and Dynamic Compressive Behavior of Gum Metal: Experiment and Constitutive Model. Metall. Mater. Trans. A.

[13] Su G., Liu Y., Xiao X., Du J., Zhang P., Shen X. (2021). Influences of Stress State, Temperature, and Strain Rate on Ductility of Pure Iron. J. Mater. Eng. Perform..

[14] Wang C.T., He Y., Guo Z., Huang X., Chen Y., Zhang H., He Y. (2021). Strain Rate Effects on the Mechanical Properties of an AlCoCrFeNi High-Entropy Alloy. Met. Mater. Int..

[15] Simoncini M., Forcellese A., Mancini E., Chiappini G., Sasso M. (2021). Experimental and numerical investigation on forming limit curves of AA6082 aluminum alloy at high strain rates. Int. J. Adv. Manuf. Technol..

[16] Sharma S., Samal M.K. (2021). Experimental Investigation of Strain-Rate- and Temperature-Dependent Mechanical Properties of SA516Gr.70 Steel and Development of an Appropriate Material Model. J. Mater. Eng. Perform..

[17] An H.M., Liu L. (2019). Numerical study of dynamic behaviors of concrete under various strain rates. Arch. Civ. Eng..

[18] Hu S., Tang H., Han S. (2021). Energy Absorption Characteristics of PVC Coarse Aggregate Concrete under Impact Load. Int. J. Concr. Struct. Mater..

[19] Wu Z., Zhang J., Yu H., Fang Q., Chen L., Yue C. (2021). Experimental and mesoscopic investigation on the dynamic properties of coral aggregate concrete in compression. Sci. China Technol. Sci..

[20] He Y., Gao M., Xu D., Yu X. (2021). Influence of Sub-zero Temperatures on the Dynamic Behaviour of Foam Concrete with Sand. KSCE J. Civ. Eng..

[21] Wang T., Song Z., Yang J., Zhang Q., Cheng Y. (2021). A Study of the Dynamic Characteristics of Red Sandstone Residual Soils Based on SHPB Tests. KSCE J. Civ. Eng..

[22] Yu L., Zhang T., Zhu Z., Su H., Fan P., Wang Y. (2021). Physical and dynamic mechanical behaviors of marble after heat treatment in quasi-vacuum and air-filled environments. J. Cent. South Univ..

[23] Huang B., Fu S., Xiao Y. (2021). Uniaxial Compressive Behavior of Granite at High Strain Rates. Rock Mech. Rock Eng..

[24] Ke B., Zhang C., Liu C., Ding L., Zheng Y., Li N., Wang Y., Lin H. (2021). An experimental study on characteristics of impact compression of freeze–thawed granite samples under four different states considering moisture content and temperature difference. Environ. Earth Sci..

[25] Majedi M.R., Afrazi M., Fakhimi A. (2021). A Micromechanical Model for Simulation of Rock Failure under High Strain Rate Loading. Int. J. Civ. Eng..

[26] Wang S., Xiong X., Liu Y., Zhou J., Du K., Cui Y., Khandelwal M. (2021). Stress–strain relationship of sandstone under confining pressure with repetitive impact. Geomech. Geophys. Geo-Energy Geo-Resour..

[27] Zhu Q., Li D., Han Z., Xiao P., Li B. (2021). Failure characteristics of brittle rock containing two rectangular holes under uniaxial compression and coupled static-dynamic loads. Acta Geotech..

[28] Zhao Z., Wu B., Zhang Z., Yu W. (2021). Impact strength characteristics of granite under combined dynamic and static loading conditions. Arab. J. Geosci..

[29] Zhao H., Liu C., Zhang J., Ge L. (2021). Breakage behavior of gravel rock particles under impact force. Comput. Part. Mech..

[30] Li M., Mao X., Cao L., Pu H. (2017). Influence of Heating Rate on the Dynamic Mechanical Performance of Coal Measure Rocks. Int. J. Geomech..

[31] Xu J., Kang Y., Wang Z., Wang X. (2020). Dynamic Mechanical Behavior of Granite under the Effects of Strain Rate and Temperature. Int. J. Geomech..

[32] Huang J., Xu S., Hu S. (2013). Effects of grain size and gradation on the dynamic responses of quartz sands. Int. J. Impact Eng..

[33] Luo H., Cooper W.L., Lu H. (2014). Effects of particle size and moisture on the compressive behavior of dense Eglin sand under confinement at high strain rates. Int. J. Impact Eng..

[34] Bragov A.M., Kotov V.L., Lomunov A.K., Sergeichev I.V. (2004). Measurement of the Dynamic Characteristics of Soft Soils Using the Kolsky Method. J. Appl. Mech. Tech. Phys..

[35] Song B., Chen W., Luk V. (2009). Impact compressive response of dry sand. Mech. Mater..

[36] Frew D.J., Forrestal M.J., Chen W. (2002). Pulse shaping techniques for testing brittle materials with a split hopkinson pressure bar. Exp. Mech..

[37] Bragov A.M., Grushevsky G.M., Lomunov A.K. (1996). Use of the Kolsky method for confined tests of soft soils. Exp. Mech..

[38] Charlie W., Ross C., Pierce S. (1990). Split-Hopkinson Pressure Bar Testing of Unsaturated Sand. Geotech. Test. J..

[39] Luo H., Lu H., Cooper W.L., Komanduri R. (2011). Effect of Mass Density on the Compressive Behavior of Dry Sand under Confinement at High Strain Rates. Exp. Mech..

[40] Parab N.D., Claus B., Hudspeth M.C., Black J.T., Mondal A., Sun J., Fezzaa K., Xiao X., Luo S.N., Chen W. (2014). Experimental assessment of fracture of individual sand particles at different loading rates. Int. J. Impact Eng..

[41] Junyu H., Songlin X., Shisheng H. (2014). Influence of particle breakage on the dynamic compression responses of brittle granular materials. Mech. Mater..

[42] Martin B.E., Chen W., Song B., Akers S.A. (2009). Moisture effects on the high strain-rate behavior of sand. Mech. Mater..

[43] Wils L., Van Impe P.O., Haegeman W. (2015). One-dimensional compression of a crushable sand in dry and wet conditions. Conference: Geomechanics from Micro to Macro.

[44] Sobczyk K., Kruszka L., Chmielewski R., Rekucki R. (2021). Performance characteristics of Hopkinson’s set-up pneumatic launcher. Acta Polytech..

[45] Zhu Z.W., Tang W.R., Kang G.Z. (2021). Dynamic Deformation of Frozen Soil at a High Strain Rate: Experiments and Damage-Coupled Constitutive Model. Acta Mech. Solida Sin..

[46] Ma D., Xiang H., Ma Q., Kaunda E.E., Huang K., Su Q., Yao Z. (2021). Dynamic Damage Constitutive Model of Frozen Silty Soil with Prefabricated Crack under Uniaxial Load. J. Eng. Mech..

[47] Chai X.J., Deng K., He C.F., Xiong Y.F. (2021). Laboratory Model Tests on Consolidation Performance of Soil Column with Drained-Timber Rod. Adv. Civ. Eng..

[48] Ma B., Hu Z., Li Z., Cai K., Zhao M., He C., Chen Q., Chen B., Huang H. (2021). A Three-Section-Settlement Calculation Method for Composite Foundation Reinforced by Geogrid-Encased Stone Columns. Adv. Civ. Eng..

[49] Nurdin S., Sawada K., Moriguchi S. (2019). Design Criterion of Reinforcement on Thick Soft Clay Foundations of Traditional Construction Method in Indonesia. MATEC Web of Conferences.

[50] Olson S.J. Eisenhower Bridge North Abutment and Approach Settlement: A Case History of Timber Pile Downdrag and Comparative Downdrag Effect on Steel Piles. Proceedings of the Geo-Congress 2020: University of Minnesota 68th Annual Geotechnical Engineering Conference.

[51] Bragov A.M., Igumnov L.A., Konstantinov A.Y., Kruszka L., Lamzin D.A., Lomunov A.K. (2021). Methodological aspects of testing brittle materials using the split Hopkinson bar technique. Strain.

[52] Ryzińska G., Gieleta R. (2020). Effect of Test Velocity on the Energy Absorption under Progressive Crushing of Composite Tubes. Adv. Sci. Technol. Res. J..

[53] Miedzińska D., Gieleta R., Małek E. (2020). Experimental study of strength properties of SLA resins under low and high strain rates. Mech. Mater..

[54] Jiang S., Shen L., Guillard F., Einav I. (2021). The effect of cement material properties on the fracture patterns developing within cement-covered brittle sphere under impact. Acta Geotech..

[55] Zhang S., Wang W., Liu K., Gong S., Li D., Li H. (2021). Dynamic behavior of coal under true triaxial prestressed by using a Hopkinson bar. Arab. J. Geosci..

[56] Chen J., Tao W., Pang S. (2021). Impact Testing of 3D Re-Entrant Honeycomb Polyamide Structure Using Split Hopkinson Pressure Bar. Appl. Sci..

[57] Jakkula P., Ganzenmueller G., Beisel S., Hiermaier S. (2021). Investigating slow shock in low-impedance materials using a direct impact Hopkinson bar setup. EPJ Web of Conferences.

[58] Deng Y.J., Chen H., Chen X.W., Yao Y. (2021). Dynamic failure behaviour analysis of TiB2-B4C ceramic composites by split Hopkinson pressure bar testing. Ceram. Int..

[59] Ishihara K. (1996). Soil Behavior in Earthquake Geotechnics. Oxford Engineering Science Series.

[60] Suescun-Florez E., Omidvar M., Iskander M., Bless S. (2015). Review of High Strain Rate Testing of Granular Soils. Geotech. Test. J..

[61] Field J.E., Walley S.M., Proud W.G., Goldrein H.T., Siviour C.R. (2004). Review of experimental techniques for high rate deformation and shock studies. Int. J. Impact Eng..

[62] Blitterswyk J., Fletcher L., Pierron F. (2017). Characterisation of the Interlaminar Properties of Composites at High Strain Rates: A Review. Adv. Exp. Mech..

[63] Siviour C.R., Jordan J.L. (2016). High Strain Rate Mechanics of Polymers: A Review. J. Dyn. Behav. Mater..

[64] Gray G.T. (2012). High-Strain-Rate Deformation—Mechanical Behavior and Deformation Substructures Induced. Annu. Rev. Mater. Res..

[65] Omidvar M., Iskander M., Bless S. (2012). Stress-strain behavior of sand at high strain rates. Int. J. Impact Eng..

[66] Mishra S., Chakraborty T., Basu D. (2015). High Strain Rate Stress-Strain Response of Soils—A Review. Jpn. Geotech. Soc. Spec. Publ..

[67] Siviour C., Kendall M.J. (2014). Understanding High Rate Behavior through Low Rate Analog. Materials Science, Final Report.

[68] Prusty G.B., Banerjee A. (2020). Structure–Property Correlation and Constitutive Description of Structural Steels during Hot Working and Strain Rate Deformation. Materials.

[69] Pająk M. Dynamic response of SFRC under different strain rates—An overview of test results. Proceedings of the 7th International Conference Analytical Models and New Concepts in Concrete and Masonry Structures.

[70] Sobczyk K., Chmielewski R., Kruszka L. (2020). The concept of experimental research on the behavior of sand cover material for protective shelters for civilians. Saf. Eng. Anthropog. Objects.

[71] Chmielewski R., Kruszka L., Rekucki R., Sobczyk K. (2021). Experimental investigation of dynamic behavior of silty sand. Arch. Civ. Eng..

[72] Bragov A.M., Balandin V.V., Igumnov L.A., Kotov V.L., Kruszka L., Lomunov A.K. (2018). Impact and penetration of cylindrical bodies into dry and water-saturated sand. Int. J. Impact Eng..

[73] Wang S., Shen L., Maggi F., El-Zein A., Nguyen G.D., Zheng Y., Zhang H., Chen Z. (2018). Influence of dry density and confinement environment on the high strain rate response of partially saturated sand. Int. J. Impact Eng..

[74] De Cola F., Pellegrino A., Glößner C., Penumadu D., Petrinic N. (2018). Effect of Particle Morphology, Compaction, and Confinement on the High Strain Rate Behavior of Sand. Exp. Mech..

[75] Barr A.D., Clarke S.D., Tyas A., Warren J.A. (2018). Effect of Moisture Content on High Strain Rate Compressibility and Particle Breakage in Loose Sand. Exp. Mech..

[76] Lv Y., Liu J., Xiong Z. (2019). One-dimensional dynamic compressive behavior of dry calcareous sand at high strain rates. J. Rock Mech. Geotech. Eng..

[77] Lv Y., Wang Y., Zuo D. (2019). Effects of particle size on dynamic constitutive relation and energy absorption of calcareous sand. Powder Technol..

[78] Wen S., Zhang C., Chang Y., Hu P. (2019). Dynamic compression characteristics of layered rock mass of significant strength changes in adjacent layers. J. Rock Mech. Geotech. Eng..

[79] Xiao Y., Yuan Z., Chu J., Liu H., Huang J., Luo S.N., Wang S., Lin J. (2019). Particle breakage and energy dissipation of carbonate sands under quasi-static and dynamic compression. Acta Geotech..

[80] Ma D., Ma Q., Yao Z., Yuan P., Zhang R. (2019). Dynamic Mechanical Properties and Failure Mode of Artificial Frozen Silty Clay Subject to One-Dimensional Coupled Static and Dynamic Loads. Adv. Civ. Eng..

[81] Varley L., Rutherford M.E., Zhang L., Pellegrino A. (2020). The Mechanical Response of Wet Volcanic Sand to Impact Loading, Effects of Water Content and Initial Compaction. J. Dyn. Behav. Mater..

[82] Dong K., Ren H., Ruan W., Ning H., Guo R., Huang K. (2020). Study on strain rate effect of coral sand. Explos. Shock. Waves.

[83] Zhao Z., Qiu Y., Zi M., Xing H., Wang M. (2020). Experimental study on dynamic compression of unsaturated calcareous sand. Explos. Shock. Waves.

[84] Lv Y., Li X., Wang Y. (2020). Particle breakage of calcareous sand at high strain rates. Powder Technol..

[85] Zhang F., Zhu Z., Fu T., Jia J. (2020). Damage mechanism and dynamic constitutive model of frozen soil under uniaxial impact loading. Mech. Mater..

[86] Zhu Z., Fu T., Zhou Z., Cao C. (2021). Research on Ottosen constitutive model of frozen soil under impact load. Int. J. Rock Mech. Min. Sci..

[87] Li T., Li G., Ding Y., Kong T., Liu J., Zhang G., Zhang N. (2021). Impact response of unsaturated sandy soil under triaxial stress. Int. J. Impact Eng..

[88] Li B., Zhu Z., Ning J., Li T., Zhou Z. (2021). Viscoelastic–plastic constitutive model with damage of frozen soil under impact loading and freeze–thaw loading. Int. J. Mech. Sci..

[89] Jia J., Tang H., Chen H. (2021). Dynamic Mechanical Properties and Energy Dissipation Characteristics of Frozen Soil under Passive Confined Pressure. Acta Mech. Solida Sin..

[90] Semblat J.F., Luong P., Gary G. (1999). 3D-Hopkinson Bar: New Experiments for Dynamic Testing on Soils. Soil Found..

[91] Martin B.E., Kabir M.E., Chen W. (2013). Undrained high-pressure and high strain-rate response of dry sand under triaxial loading. Int. J. Impact Eng..

[92] Kabir M.E., Chen W.W. (2011). Dynamic Triaxial Test on Sand. Dyn. Behav. Mater..

[93] Liu K., Zhang Q.B., Wu G., Li J.C., Zhao J. (2019). Dynamic Mechanical and Fracture Behaviour of Sandstone Under Multiaxial Loads Using a Triaxial Hopkinson Bar. Rock Mech. Rock Eng..

[94] Casante G., Santamarina J.C. (1996). Interparticle Contact Behavior and Wave Propagation. J. Geotech. Eng..

[95] Senetakis K., Payan M., Li H., Zamanian M. (2021). Nonlinear stiffness and damping characteristics of gravelly crushed rock: Developing generic curves and attempting multi-scale insights. Transp. Geotech..

